# Reducing variability in nasal surgery outcomes through computational fluid dynamics and advanced 3D virtual surgery techniques

**DOI:** 10.1016/j.heliyon.2024.e26855

**Published:** 2024-02-28

**Authors:** M.A. Burgos, Lina Rosique, F. Piqueras, C. García-Navalón, M.A. Sevilla-García, D. Hellín, F. Esteban

**Affiliations:** a Department of Ingeniería Térmica y de Fluidos, Universidad Politécnica de Cartagena, Spain; b Department of Surgery, School of Medicine, University of Seville, Spain; c Department of Otolaryngology, Hospital Universitario Virgen del Rocío, Seville, Spain; d Department of Otolaryngology, Hospital General Universitario Morales Meseguer, Murcia, Spain; e Department of Otolaryngology, Consorcio Hospital General Universitario de Valencia, Spain; f Department of Otolaryngology, Hospital Clínico Universitario Virgen de la Arrixaca, Murcia, Spain

**Keywords:** Nasal surgery variability, Computational fluid dynamics (CFD), 3D virtual surgery, Otolaryngology, Improved patient outcomes, Nasal airflow assessment, Nasal obstruction treatment, Personalized surgical planning

## Abstract

**Objectives:**

This study aims to delineate the specific impact of using computational fluid dynamics (CFD) and 3D virtual surgery techniques in otolaryngology surgery, focusing on their roles in enhancing the precision of nasal surgery and optimizing future patient outcomes. The central objective was to assess whether these advanced technologies could reduce variability in surgical approaches and decision-making among specialists, thereby improving the consistency and efficacy of patient care in cases of nasal obstruction.

**Methods and results:**

Our methodology involved a detailed analysis of pre- and post-operative scenarios using CFD feedback. Six otolaryngologists participated, employing virtual surgery techniques on two patients with diagnosed nasal obstruction. The CFD analysis focused on quantifying key airflow parameters: right nasal flow rate (QR), left nasal flow rate (QL), flow symmetry (Ф), and bilateral nasal resistance (R). These parameters were meticulously compared before and after the application of CFD feedback to evaluate changes in surgical planning and outcomes. Quantitative analysis revealed a notable decrease in the standard deviation of the measured parameters among the specialists post-CFD feedback, indicating reduced variability in surgical approaches. Specifically, for Patient #1 the standard deviation for QR values dropped from 0.694 L/min to 0.602 L/min, and for QL values from 0.676 L/min to 0.584 L/min, and for Patient #2, the standard deviation for QR values decreased from 2.204 L/min to 0.958 L/min, and for QL values from 2.295 L/min to 1.014 L/min. Moreover, the variability range, represented by the differences between the maximum and minimum values for Ф and R, diminished significantly. Post-operative average values for all parameters showed a convergence towards ideal basal levels, suggesting a more uniform and effective surgical strategy across different surgeons.

**Conclusions:**

Both integration of CFD and 3D virtual surgery techniques in otolaryngology can substantially reduce variability in surgical planning and decision-making, ultimately leading to improved patient outcomes. These advanced tools have the potential to standardize the diagnosis and treatment of nasal pathologies, contributing to more effective and consistent care. Future research in this area should focus on larger patient cohorts and further exploration of the potential benefits and applications of CFD and virtual surgery in otolaryngology.

## 1 Introduction

Septoplasty and turbinoplasty are among the most frequent surgical procedures performed by head and neck surgeons, as nasal septal deviation and turbinate hypertrophy are one of the most common causes of nasal obstruction, and yet patients are frequently listed for surgery to both the septum and turbinates without any objective assessment of their airway being performed [1,2]. Out comes are rated using subjective scales of nasal obstruction (NOSE), or visual analogic scales (VAS) [3]. However, years of prospective trials in septoplasty suggest widely differing degrees of success in long-term patient-reported outcomes. Clinical assessment via history and physical exam has long been the gold standard in identifying patients for whom septoplasty is indicated, although it has been demonstrated that it has not been sufficient to ensure consistently good outcomes for all patients [4]. Several factors contribute to the lack of consistent results, such as the reliance on subjective data, personal knowledge, and experience. Besides, there significant evidence in the literature regarding the relative correlation between objective findings and patients’ perception of nasal obstruction, even with patients with documented relevant nasal deviation without any nasal complaint [5]. Although many studies have demonstrated a significant correlation between nasal patency, assessed by visual analogue score or questionnaire, and nasal resistance, as measured by anterior rhinomanometry or acoustic rhinometry, other studies, however, have found no such correlation between objective and subjective assessment (see for review Roblin & Eccles, 2002) [6]. Some authors even stated that effectiveness of septoplasty in relieving nasal obstruction in patients with deviated nasal septum has not been proven [7,8]. Emerging techniques, such as Computational Fluid Dynamics (CFD) and virtual surgery, have shown promise in addressing this variability and improving patient outcomes [9].

CFD is a powerful simulation tool used to model fluid flow using the Navier-Stokes equations [10]. CFD can provide valuable insights into the patient's nasal respiratory system function, such as pressure distribution, velocity, and turbulence [[11], [12], [13], [14]]. CFD simulations can also predict postoperative outcomes and guide surgical decisions, helping surgeons plan surgeries more effectively [15].

Virtual surgery, another emerging technique, utilizes high-resolution imaging such as computed tomography (CT) or magnetic resonance imaging (MRI) to create a three-dimensional (3D) representation of the nose and nasal cavities. This 3D representation enables the surgeon to simulate and evaluate different surgical strategies before performing the actual surgery [15]. Virtual surgery also allows for computer-assisted surgical navigation, which helps surgeons operate with greater precision and accuracy. However, challenges such as cost, complexity, and the need for further research persist before these techniques become standard practice in nasal surgery. The integration of CFD and virtual surgery into surgical practice requires specialized equipment, software, and trained personnel, potentially limiting widespread adoption.

Several studies have investigated the use of CFD and virtual surgery in nasal surgery. For instance, Frank-Ito and Garcia (2021) [16] used CFD to study the impact of nasal surgery on airflow patterns and nasal resistance, providing valuable information for surgical planning. Moghaddam et al. (2020) [9] found that virtual surgery allows to assess surgical outcomes in a patient undergoing functional nasal surgery.

The integration of CFD and virtual surgery holds the potential to enhance both education and communication between surgeons and patients. By displaying a visual representation of nasal airflow and virtual surgery results, surgeons can better explain treatment options and anticipated outcomes, reducing anxiety and increasing patient satisfaction. These techniques can also help reduce surgical risks and improve safety, as the practice of different approaches in a virtual environment before performing the actual surgery minimizes errors and increases surgical accuracy.

The objective of this study is to examine the potential of CFD and 3D virtual surgery in reducing the variability of nasal surgery outcomes and to investigate the impact of incorporating CFD analysis and 3D virtual surgery techniques on the variability of nasal surgery outcomes. By comparing surgical outcomes before and after the implementation of these technologies, we will analyze if integration of CFD and 3D virtual surgery can significantly reduce variability in otolaryngology, ultimately leading to improved patient care and more predictable surgical results.

## 2 Methods

### 2.1 Patient selection and data collection

In this study, we analyzed Two patients who presented with nasal obstruction requiring surgical intervention were analyzed. Both patients underwent an extensive preoperative assessment. Data collection involved a retrospective review, with a detailed medical history, physical examination, and diagnostic imaging studies such as computed tomography (CT) scans of the nasal cavity. Data were anonymized to ensure patient confidentiality and adhere to ethical guidelines for research involving human subjects. The two patients featured in this study were randomly selected from a larger group of patients participating in a clinical trial at Hospital General Universitario Morales Meseguer in Murcia and Consorcio Hospital General Universitario in Valencia, both located in Spain. The clinical trial received approval from the Ethics Committees of both hospitals. These patients were chosen randomly to ensure a diverse and representative sample of those requiring intervention within the context of the broader trial.

Table 1 provides a summary of the data of the two patients, detailing their gender, age, and the characteristics of their CT scan.

**Table 1:** Summary of the data for each patient, detailing the characteristics of their CT scan (Dimensions and Slide Thickness), their gender and, age.

	CT Scan Dimensions	CT Scan Slice Thickness	Gender	Age
**Patient #1**	528 × 528 × 360	0.6	M	28
**Patient #2**	512 × 512 × 447	0.6	F	52

For every patient, six experienced otolaryngologists performed a virtual surgery using a 3D model of the patient's nasal cavity, which was reconstructed from the CT scans. These virtual surgeries were conducted without prior knowledge of the computational fluid dynamics (CFD) results. After the initial round of virtual surgeries, the otolaryngologists were provided with the CFD results, which included information on the nasal airflow patterns and resistance. The otolaryngologists then performed a second round of virtual surgeries, this time taking into consideration the CFD results.

CT scans from the patients were analyzed using Flowgy© software [17], a comprehensive tool for conducting Computational Fluid Dynamics (CFD) studies and virtual surgery on 3D models. The software covers all necessary steps, including segmentation, computational meshing, resolving airflow using CFD techniques, and post-processing the solution. We followed the established workflow from previous articles where the parameters were first defined [15,18,19].

Data from these virtual surgeries, including the volumetric flow rates (QR and QL) and the dimensionless parameters Ф (symmetry of the flow) and R (bilateral nasal resistance), were collected and analyzed [20]. Additionally, we obtained baseline data for each patient, which represented the nasal cavity's condition before any virtual surgery. This baseline data served as a reference for comparing the outcomes of the virtual surgeries.

### 2.2 Ethical considerations

All procedures performed in this study involving human participants were in accordance with the ethical standards of the Ethical Committee at Polytechnic University of Cartagena and with the 1964 Helsinki declaration and its later amendments or comparable ethical standards. This study was reviewed and approved by the Ethical Committee at Polytechnic University of Cartagena, receiving the ethics approval number CEI23_005. The ethics committee's review and approval ensure that all research activities conform to international guidelines for human research ethics and safeguard the rights, safety, and well-being of all participants.

### 2.3 CFD analysis

The computational fluid dynamics (CFD) analysis in this study was conducted using state-of-the-art software and numerical methods. The 3D models of the patients' nasal cavities were imported into the CFD software, where the airflow patterns, pressure distributions, and other relevant fluid properties were simulated and analyzed. The CFD simulations were performed under steady-state conditions and with the inhalation phase of the breathing cycle.

Utilizing Flowgy© software, the nasal airflow of two patients was resolved through OpenFOAM [10], a widely-used open-source CFD solver. OpenFOAM's laminar solver was employed to analyze the steady-state, compressible, and laminar airflow within the patients' nasal cavities. To ensure equal flow rates in both nasal cavities and maintain a realistic breathing rate of approximately 15 L per minute, a pressure drop was imposed between the atmospheric pressure and the nasopharyngeal region. This approach is consistent with the majority of similar simulation studies and ensures a calm, steady breathing pattern [21,22].

**Fig. 1:**
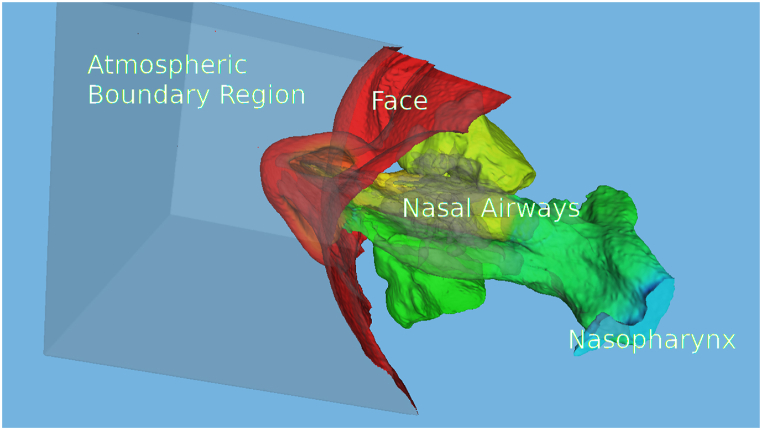
Detailed 3D representation of the computational domain, highlighting the patient's face, atmospheric boundary region, and key components of the nasal airways and nasopharynx for accurate airflow simulation.

**Fig. 2:**
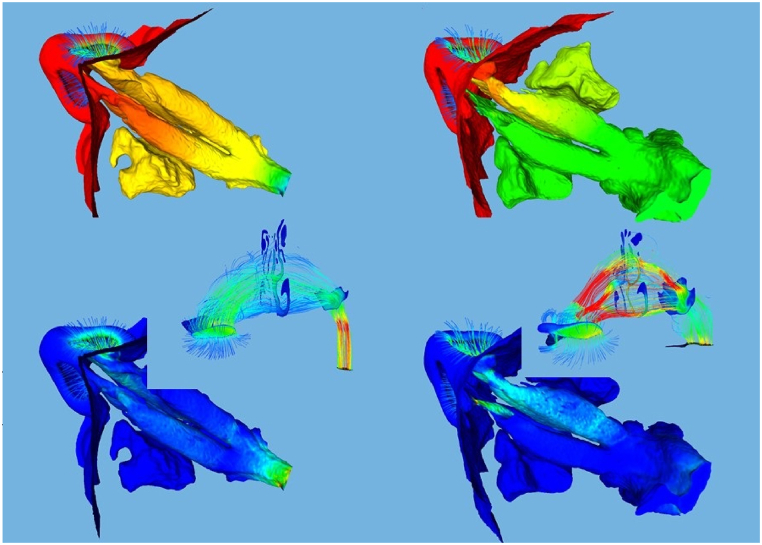
Computational Models and Flow Dynamics for Patients: This figure illustrates the computational fluid dynamics models for two distinct patients. On the left, the model of Patient #1 is depicted, while on the right, we feature the model of Patient #2. The upper panels of the figure highlight the pressure fields within the nasal cavities. The lower panels focus on Wall Shear Stress (WSS). A detailed inset showcases streamline patterns overlaid with the velocity field. All figures and fluid fields correspond to the Basal CFD simulation.

**Fig. 3:**
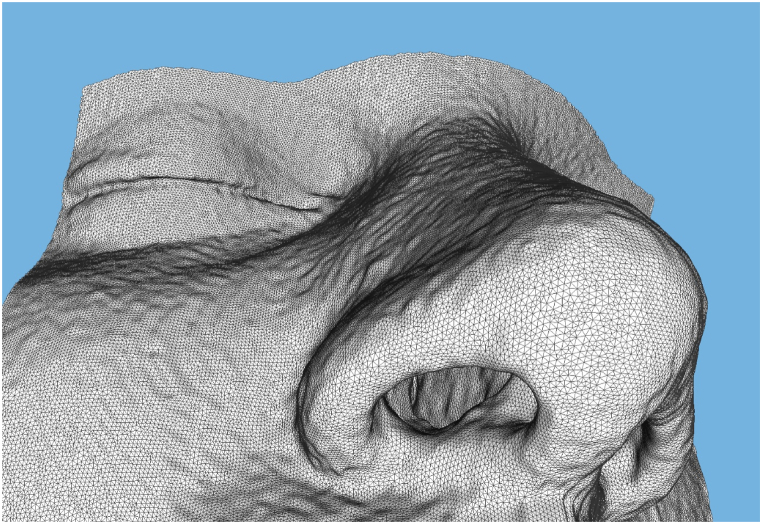
Detailed View of the Computational Mesh Surface. This figure illustrates the surface mesh used in our Computational Fluid Dynamics (CFD) analysis. The mesh, comprising approximately 9 million tetrahedral elements.

In both cases, the simulation was conducted during inspiration. The laminar flow condition within the nasal cavities is ensured by maintaining the Reynolds number well below the critical threshold of 2000. The Reynolds number is a dimensionless quantity that characterizes the flow regime, providing insight into the balance between inertial and viscous forces in the fluid. When the Reynolds number is lower than the threshold, the flow remains laminar, while values above the threshold indicate the onset of turbulence. To calculate the Reynolds number (Re) for the nasal airflow, the hydraulic radii of the nasal orifices and the choana are utilized as reference lengths. The hydraulic radius (R_h_) is defined as the ratio of the flow area (A) to the wetted perimeter (P), expressed mathematically as R_h_ = A/P. To enhance the accuracy of the simulation, the patient's face geometry was also incorporated into the computational domain (Fig. 1). This addition enabled a more precise representation of the air intake into the nasal cavity, better reflecting real-world conditions. To validate the computational fluid dynamics (CFD) simulations, a mesh convergence analysis was conducted for both patients. Consequently, meshes consisting of around 9 million tetrahedra were used for the pre- and post-surgery nasal geometries for both patients. In Fig. 2, we present the computational models of the two patients studied, showcasing the intricate details captured by our analyses. On the left, we display the model for Patient #1, and on the right, that of Patient #2. These models are illustrated with two distinct fields: the pressure fields are depicted in the upper panels, highlighting the differential pressures across the nasal cavities, while the Wall Shear Stress (WSS) is visualized in the lower panels, providing insights into the mechanical forces exerted by airflow on the nasal walls. Additionally, a detailed view of the streamline patterns, accompanied by the velocity field, is provided. All fluid fields correspond to the Basal CFD simulation. Fig. 3 presents a detailed view of the computational surface mesh.

The CFD analysis provided valuable insights into the flow characteristics within the nasal cavity, such as the distribution of airflow between the left and right nostrils, the presence of turbulent flow regions, and the overall resistance to airflow. These insights allowed for a better understanding of the patients' preoperative nasal function and helped inform the otolaryngologists' surgical planning in the second round of virtual surgeries.

### 2.4 3D virtual surgery techniques

The Virtual surgeries were conducted using the integrated module within the Flowgy© software, which enables 3D virtual surgery to be performed directly on the patient's 3D model in an endoscopic manner, closely simulating the real-world surgical environment that physicians encounter in the operating room. This virtual surgery module is synchronized with the patient's CT scan data, allowing the surgeon to perform the procedure in a synchronized manner on both the CT scan images and the 3D model.

Using the mouse pointer as a virtual scalpel, the surgeon can make precise alterations to the 3D model, simulating the effects of the surgical procedure. These modifications to the 3D model are simultaneously reflected in the corresponding CT scan images, providing an integrated and cohesive visual representation of the surgical outcome. This innovative approach grants the medical professional the ability to visualize the impact of their surgical decisions in real-time, offering a more comprehensive understanding of the procedure's consequences.

This synchronization between the 3D model and the CT scan images not only enhances the surgical planning process but also serves as a valuable educational tool, providing a platform for surgeons to refine their skills and techniques in a controlled, virtual environment. By incorporating the patient's unique facial geometry and simulating the endoscopic perspective, the software ensures that the virtual surgery experience closely replicates the conditions of an actual operating room, ultimately leading to better patient outcomes and improved surgical proficiency.

**Fig. 4:**
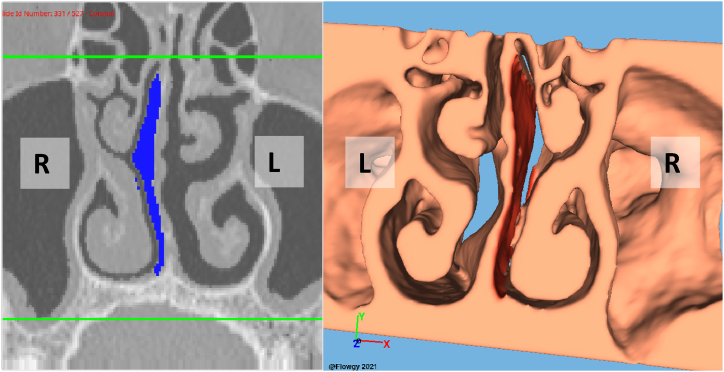
This figure illustrates the 3D model employed for virtual surgery on Patient #1, alongside the corresponding virtual surgical modifications overlaid on the coronal plane of the CT scan.

**Fig. 5:**
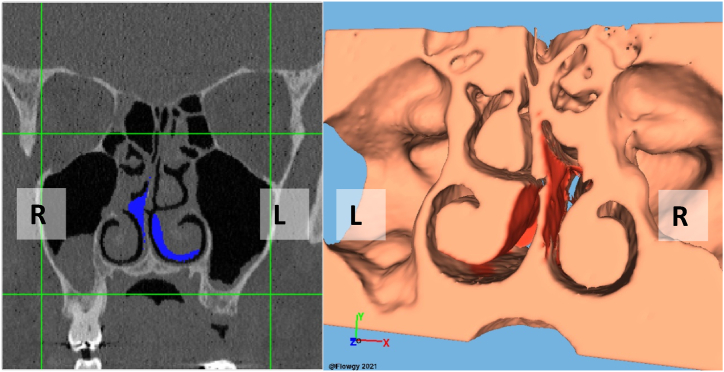
This figure illustrates the 3D model employed for virtual surgery on Patient #2, alongside the corresponding virtual surgical modifications overlaid on the coronal plane of the CT scan.

In Fig. 4, Fig. 5, we present a detailed three-dimensional representation of the models used for virtual surgery on each patient involved in this study. These visualizations are rendered using state-of-the-art OpenGL graphics libraries, which provide efficient and high-quality rendering capabilities for 3D graphics. The figure showcases the integration of advanced computer graphics techniques, such as real-time shading and advanced texturing, to achieve a lifelike representation of the patient's anatomy.

On the right side of the figure, we see the 3D model on which the virtual surgery is performed. This model has been meticulously constructed using the patient's CT scan data and employs advanced meshing techniques to ensure an accurate representation of the patient's nasal structures. The model allows the otorhinolaryngologist specialist medical professional to perform virtual surgery using an intuitive interface, simulating the surgical process with high fidelity.

On the left side of the figure, we can observe the coronal view of the CT scan, which displays the modifications resulting from the virtual surgery. This synchronized visualization demonstrates the impact of the surgeon's actions on the patient's anatomy, providing valuable insights into the procedure's effects. The combination of the 3D model and the corresponding CT scan images delivers a comprehensive and interactive platform for surgical planning and practice, incorporating cutting-edge computer graphics and visualization techniques to enhance the overall virtual surgery experience.

The Virtual surgeries consisted in various surgical techniques commonly used in otolaryngology, such as septoplasty, turbinate reduction, nasal valve surgery and sinus surgery. By simulating these procedures in a virtual environment, the otolaryngologists were able to evaluate the potential impact of their surgical decisions on the patients' nasal airflow and resistance, thus informing their surgical planning and potentially improving patient outcomes.

### 2.5 Comparison of outcomes before and after CFD feedback

To assess the impact of CFD feedback on the outcomes of the virtual surgeries, we compared the data obtained before and after the otolaryngologists were provided with the CFD results. This comparison involved an analysis of the volumetric flow rates (QR and QL), as well as the dimensionless parameters Ф and R, for each patient and each round of virtual surgery.

Our analysis revealed noticeable differences in the outcomes of the virtual surgeries performed before and after the CFD feedback was provided. In particular, we observed a reduction in the variability of the flow symmetry parameter Ф and the bilateral nasal resistance parameter R, suggesting that the incorporation of CFD results into the surgical planning process led to more consistent and potentially more effective surgical outcomes.

Furthermore, we conducted a statistical analysis to determine the significance of these differences. This statistical analysis allowed us to determine if the observed improvements in the virtual surgery outcomes were statistically significant or could have occurred by chance.

In addition to the quantitative analysis, we also gathered qualitative feedback from the participating otolaryngologists. This feedback provided valuable insights into the surgeons' experiences and perceptions of the usefulness of the CFD results in their surgical planning.

## 3 Results

### 3.1 Surgical procedures in every patient

Tables offer insight into the surgical decisions made by six different surgeons (ENTs) before and after receiving Computational Fluid Dynamics (CFD) feedback for two patients. Table 2 and Table 3 illustrate the adjustments made by the surgeons based on the CFD simulation data.

**Table 2:** Surgical interventions performed by six ENT surgeons on patient #1 before and after receiving Computational Fluid Dynamics (CFD) feedback.

ENT #	Patient #1 Pre-CFD feedback	Patient #1 Post-CFD feedback
**ENT 1**	Anterior septoplasty work (Cottle area 2) and septal spur resection to the right nasal cavity and right turbinoplasty.	Minor revision of previous surgery.
**ENT 2**	Septoplasty correcting the septal crest obstructing areas III and IV and a partial reduction of the right inferior turbinate head.	Increased small anterior septal deviation and completed volumetric reduction of both inferior nasal turbinates.
**ENT 3**	Valvuloplasty + right turbinoplasty + endoscopic right septoplasty (resected chondrovomerian spur).	Anterosuperior and premaxilla septoplasty, bilateral turbinoplasty.
**ENT 4**	Surgery to correct the posterior septal crest in area III-IV and a reduction of both turbinate heads.	Greater septal work in areas I and II, and expanding the reduction of both inferior turbinates with a decrease of their body and tail by one-third more than initially worked.
**ENT 5**	Extended septoplasty correcting the right nasal passage	NO Changes
**ENT 6**	Septoplasty and right turbinate reduction.	Attention and better realignment in the anterior septal area (area 2).

**Table 3:** Surgical interventions performed by six ENT surgeons on patient #2 before and after receiving Computational Fluid Dynamics (CFD) feedback.

ENT #	Patient #2 Pre-CFD feedback	Patient #2 Post-CFD feedback
**ENT 1**	Septoplasty at the level of the septal spur to the right nasal cavity.	More septoplasty work is added in the right nasal cavity valve area and some in left nasal cavity.
**ENT 2**	Intense septoplasty work, correcting the dysmorphia in both nasal cavities and volumetric reduction of both inferior nasal turbinates.	Small corrections in the septum and both nasal turbinates are expanded.
**ENT 3**	Val of right septal spur, bilateral valvuloplasty, left inferior turbinoplasty	Extensive left turbinoplasty, removal of right septum and premaxilla.
**ENT 4**	Septal work to correct nasal flow, including straightening the septal deviation from area I to area IV.	NO Changes
**ENT 5**	Left valvuloplasty, septoplasty working mainly on the right side with removal of septal spur. Wide right inferior turbinoplasty	No Changes
**ENT 6**	Right removal of septal spur and right septum luxation at premaxilla, minimal inferior bilateral turbinoplasty	Extender anterior septoplaty

**Fig. 6:**
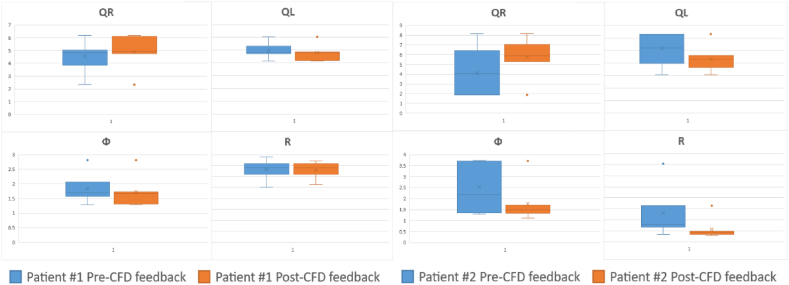
Boxplot Analysis Pre and Post CFD Feedback for Patient 1 and 2. Comparative visualization of Right Flow Rate (QR), Left Flow Rate (QL), Flow Symmetry (Ф), and Resistance (R) before and after virtual surgery guided by CFD insights.

To evaluate the influence of Computational Fluid Dynamics (CFD) feedback on the outcomes of ENT surgeries, a comprehensive statistical analysis was undertaken. The findings from this analysis are detailed in Table 4 and Table 5, and visually depicted in Fig. 6. This figure presents a comparative analysis of key metrics for both Patient 1 and Patient 2, capturing the variations in these metrics before and after virtual surgeries. Specifically, Fig. 6 comprises boxplot representations of four crucial parameters: Right Flow Rate (QR), Left Flow Rate (QL), Flow Symmetry (Ф), and Resistance (R).

**Table 4:** Patient #1 and Patient #2 Pre-CFD feedback airflow data. QR represents the airflow in the right nasal cavity (in L/min), QL represents the airflow in the left nasal cavity (in L/min), Ф is a dimensionless parameter that measures the symmetry of the airflow, and R denotes the bilateral nasal resistance. SD: standard deviation. MAX: maximum value. MIN: minimum value. Rg: range. M: sample mean.

Patient #1 Pre-CFD feedback					Patient #2 Pre-CFD feedback				
	QR (L/min)	QL (L/min)	Ф	R		QR (L/min)	QL (L/min)	Ф	R
**Basal**	2.327	12.088	2.812	7.161	**Basal**	1.877	12.653	3.719	65.594
**ENT 1**	4.644	9.823	1.759	6.908	**ENT 1**	6.402	7.952	1.284	141.685
**ENT 2**	5.038	9.439	1.626	6.588	**ENT 2**	1.872	12.656	3.724	61.032
**ENT 3**	3.847	10.617	2.063	7.784	**ENT 3**	4.136	10.315	2.151	26.273
**ENT 4**	6.201	8.326	1.290	6.213	**ENT 4**	4.055	10.419	2.194	30.258
**ENT 5**	4.831	9.649	1.696	6.824	**ENT 5**	8.148	6.14	1.354	13.181
**ENT 6**	4.959	9.439	1.572	5.018	**ENT 6**	2.267	12.276	3.366	31.05
**SD**	0.694	0.676	0.230	0.835	**SD**	2.204	2.295	0.923	43.191
**MAX**	6.201	10.617	2.063	7.784	**MAX**	8.148	12.656	3.724	141.685
**MIN**	3.847	8.326	1.290	5.018	**MIN**	1.872	6.140	1.284	13.181
**Rg**	2.354	2.291	0.773	2.766	**Rg**	6.276	6.516	2.440	128.503
**M**	4.920	9.549	1.668	6.556	**M**	4.480	9.960	2.345	50.580

**Table 5:** Patient #1 and Patient #2 Post-CFD feedback airflow data. QR represents the airflow in the right nasal cavity (in L/min), QL represents the airflow in the left nasal cavity (in L/min), Ф is a dimensionless parameter that measures the symmetry of the airflow, and R denotes the bilateral nasal resistance. SD: standard deviation. MAX: maximum value. MIN: minimum value. Rg: range. M: sample mean.

Patient #1 Post-CFD feedback					Patient #2 Post-CFD feedback				
	QR (L/min)	QL (L/min)	Ф	R		QR (L/min)	QL (L/min)	Ф	R
**Basal**	2.327	12.088	2.812	7.161	**Basal**	1.877	12.653	3.719	65.594
**ENT 1**	4.748	9.741	1.725	6.904	**ENT 1**	7.048	7.278	1.104	16.607
**ENT 2**	6.11	8.399	1.312	6.498	**ENT 2**	6.239	8.133	1.338	18.971
**ENT 3**	4.94	9.571	1.663	7.410	**ENT 3**	5.254	9.18	1.679	13.874
**ENT 4**	6.201	8.326	1.290	6.213	**ENT 4**	5.885	8.619	1.693	11.302
**ENT 5**	4.831	9.649	1.696	6.824	**ENT 5**	8.148	6.14	1.354	13.181
**ENT 6**	5.068	9.331	1.539	5.240	**ENT 6**	5.745	8.628	1.491	19.405
**SD**	0.602	0.584	0.176	0.678	**SD**	0.958	1.014	0.205	3.004
**MAX**	6.201	9.741	1.725	7.410	**MAX**	8.148	9.180	1.693	19.405
**MIN**	4.748	8.326	1.290	5.240	**MIN**	5.254	6.140	1.104	11.302
**Rg**	1.453	1.415	0.435	2.169	**Rg**	2.894	3.040	0.588	8.102
**M**	5.316	9.170	1.537	6.515	**M**	6.387	7.996	1.443	15.557

### 3.2 Pre-CFD feedback results

**Fig. 7:**
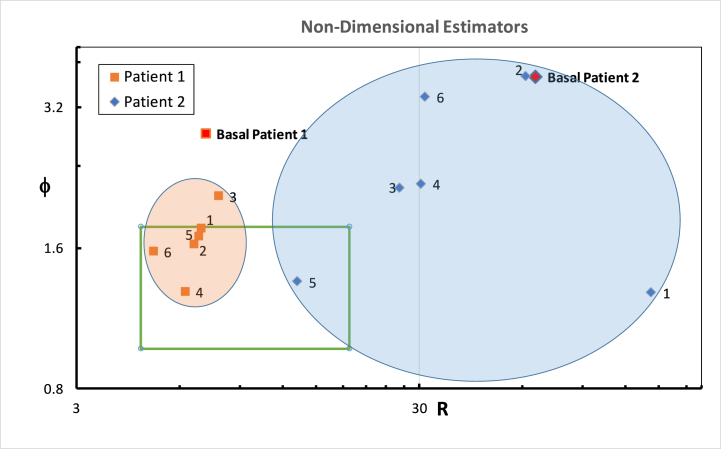
Scatter plot depicting the relationship between the Ф and R parameters for the data represents the surgical outcomes for the two patients performed by the six surgeons before receiving CFD feedback. Ф is a dimensionless parameter that measures the symmetry of the airflow, and R represents the bilateral nasal resistance. Each data point corresponds to a specific surgical outcome.

Before the CFD feedback was provided, the otolaryngologists performed virtual nasal surgery on both patients. The nasal flow metrics (QR, QL, Ф, and R) for each patient were assessed, as shown in Table 4. For Patient #1, we observed a high degree of variability in the values of Ф and R among the six ENT specialists. The standard deviation for Ф was 0.2306, with a maximum value of 2.063 and a minimum value of 1.290. Similarly, the standard deviation for R was 0.835, with a maximum value of 7.784 and a minimum value of 5.018. For Patient #2, the pre-CFD feedback values showed an even greater degree of variability. The standard deviation for Ф was 0.9232, with a maximum value of 3.7246 and a minimum value of 1.28458. The standard deviation for R was 43.19156, with a maximum value of 141.685 and a minimum value of 13.1817. Fig. 7 shows the graphical representation on a Cartesian plane of the Ф and R before the surgeons receiving CFD feedback.

### 3.3 Post-CFD feedback results

**Fig. 8:**
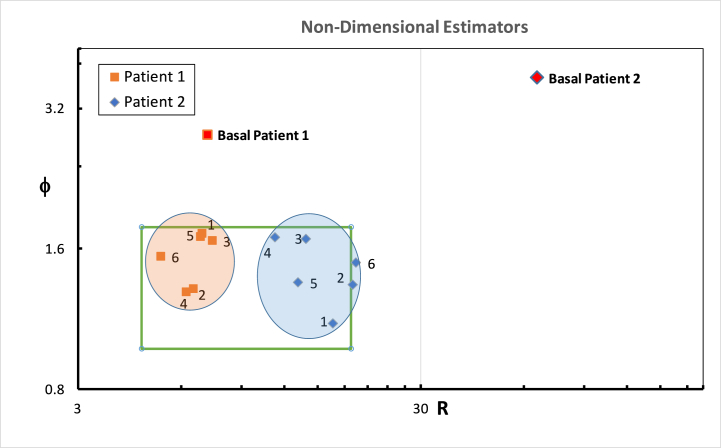
Scatter plot depicting the relationship between the Ф and R parameters for the data represents the surgical outcomes for the two patients performed by the six surgeons after the surgeons received CFD feedback. Ф is a dimensionless parameter that measures the symmetry of the airflow, and R represents the bilateral nasal resistance. Each data point corresponds to a specific surgical outcome.

Once the CFD feedback was provided to the otolaryngologists, they performed another round of virtual nasal surgery on the same patients. The nasal flow metrics (QR, QL, Ф, and R) were reassessed, as shown in Table 5. For Patient #1, we observed a reduction in the variability of the Ф and R values among the six ENT specialists. The standard deviation for Ф decreased to 0.1769, with a maximum value of 1.725 and a minimum value of 1.29. Similarly, the standard deviation for R decreased to 0.6786, with a maximum value of 7.410 and a minimum value of 5.240. For Patient #2, we observed a similar reduction in variability. The standard deviation for Ф decreased to 0.205, with a maximum value of 1.69358 and a minimum value of 1.104. The standard deviation for R decreased to 3.004, with a maximum value of 19.405 and a minimum value of 11.303. Fig. 8 shows the graphical representation on a Cartesian plane of the Ф and R after the surgeons receiving CFD feedback.

### 3.4 Comparison of QR and QL values

In Patient #1, before incorporating CFD feedback, surgeons demonstrated significant variability in surgical outcomes. The QR values ranged from 3.847 L/min to 6.201 L/min, while QL values varied between 8.326 L/min and 10.617 L/min. This variability could potentially result in postoperative complications, such as uneven airflow distribution and breathing difficulties, adversely impacting the patient's quality of life.

Upon integrating CFD feedback into the surgical planning process, surgeons exhibited a noticeable reduction in variability. The QR values ranged from 4.748 L/min to 6.201 L/min, and QL values from 8.326 L/min to 9.741 L/min.

The standard deviation for QR values dropped from 0.694 L/min to 0.602 L/min, and for QL values from 0.676 L/min to 0.584 L/min.

Patient #2 displayed a similar trend. Prior to receiving CFD feedback, the QR values among surgeons ranged from 1.872 L/min to 8.148 L/min, and QL values from 6.14 L/min to 12.656 L/min.

Incorporating CFD feedback into the surgical planning process led to a significant reduction in variability for Patient #2. The QR values ranged from 5.254 L/min to 8.148 L/min, and QL values from 6.14 L/min to 9.18 L/min. This increased consistency and precision among surgeons resulted in better airflow symmetry.

The standard deviation for QR values in Patient #2 decreased from 2.204 L/min to 0.958 L/min, and for QL values from 2.295 L/min to 1.014 L/min. These reductions in standard deviation emphasize the positive impact of CFD feedback on surgeons' ability to optimize airflow in both nasal cavities.

**Fig. 9:**
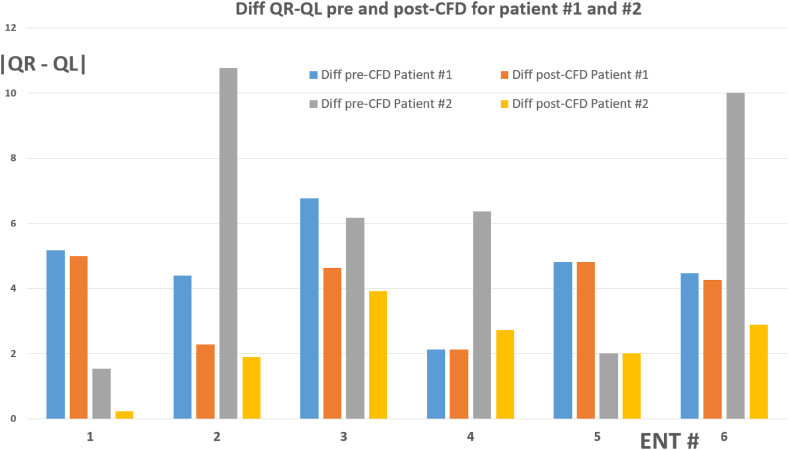
Comparative Analysis of Airflow Rate Differences between Right and Left Nasal Passages in Patients #1 and #2, Pre and Post CFD Feedback. This figure illustrates the disparities in airflow through the right and left nasal orifices for each patient, highlighting how these differences are modified before and after receiving insights from Computational Fluid Dynamics (CFD) analysis. The graph provides a clear visualization of the trend towards improved nasal flow symmetry post-CFD feedback.

In Fig. 9, we have illustrated the variations in airflow rates differences between the right and left nasal passages for each surgeon and each patient. This graph distinctly showcases the reduction in these differences achieved by each surgeon after receiving CFD feedback. The data vividly demonstrate a trend among all surgeons, across both patients, towards enhancing the symmetry of nasal airflow. This convergence towards more balanced flow rates post-feedback is indicative of the valuable role that CFD analysis plays in guiding surgeons towards more precise and symmetrical surgical outcomes.

## 4 Discussion

Patients complaining of nasal obstruction represent a very common clinical entity. It has been reported that up to 25% of the population suffers from nonallergic nasal obstruction [23]. For the general otolaryngologist, treatment of nasal obstruction is equallyfrequent. It has been reported that up to 42% of the population may have some form of septal deviation but as much as a 15% may complain of symptoms of nasal obstruction. Septoplasty was reported to be the third most commonly performed procedure in 1992 with more than 260.000 procedures completed in the United States in 2006 [4] and turbinate surgery the eighth most common procedure performed by otolaryngologists [24]. However, surgical correction of the nasal deformities as consider by the clinician does not always guarantee a successful outcome. In addition, there is little correlation between septal deviation findings on CT scans and symptoms of nasal obstruction. In the paper of Ardeshirpour et al. (2015) [25] they found a poor correlation between NOSE scores and degree of septal deviation, and furthermore, a lack of concordance between the side of the symptoms reported and the side that the clinicians considered most deviated on CT, not supporting a role for CT scans as either a clinically meaningful or necessary test to investigate uncomplicated nasal obstruction. In a recent paper from our group [5] a high incidence of nasal problems considered of clinical relevance was recorded in a group of patients who underwent mastoidectomy for cholesteatoma, and no one complained of nasal obstruction. As a result, surgeons rely mainly in their formation and experience, and this is the main goal of the current study: to investigate how two patients complaining of nasal obstruction were surgically managed by six different and experienced rhinologists, based only in the CT scan images provided.

At least in our country, there is currently a trend for less septoplasty and more turbinate surgery, the so-called ‘septum preservation surgery’ from our registrars. From one side, there is a physical reason to explain this clinical option, as it is well established by Poiseuille's Law that as little as a 10% reduction in the cross-sectional area of the nasal passage can produce a 21% increase in the airflow through the nose, and decongestion of the nose has been shown to increase the total volume of the nasal cavity by 35% [26]. Latte and Taverner (2005) [27] demonstrated that 70% of the effects on nasal resistance caused by decongestants can be achieved with external nasal dilators. These effects occur mainly in the nasal valve area and the anterior region of the inferior turbinate, and these findings are most dramatic in patients with septal deviation. As the main effect of decongestants is vasoconstriction of the inferior turbinate, and the anterior portion of the inferior turbinate is intimately associated with the region of the nasal valve area, it is quite logical to suppose, and the clinical practice agrees, that reducing the size of the turbinates can produce a significant change in nasal obstruction, even in the presence of a septal deviation. All the six surgeons in our study performed turbinate surgery in different degrees, and it is surprising how the first round of surgeries resulted in so different CFD results.

From other side, there is a feeling that septoplasty alone is not a consistent solution for the patients of nasal obstruction. Sundh and Sunnersen (2015) [28] registered that more than half of their patients reported that their symptoms remained or had worsened 34–70 months after septoplasty. Another important finding was that as many as 83 % of the patients reported nasal obstruction at long-term follow-up. Thus, it seems evident that septoplasty alone is not really efficient, and turbinoplasty emerged as low-cost, less morbidity, first-line option for patients with nasal obstruction, even before septoplasty which needs general anesthesia. In fact, a recent Consensus on Septoplasty consider turbinate reduction as an associate procedure, considering in all their conclusions septoplasty ‘with or without inferior turbinate reduction’ [29].

Today, there is no universal standard to objectively measure, report, treat, or assess subjective outcomes of nasal obstruction. It has been reported a failure rate of approximately 27–84%, 6 months to 11 years after surgery associated with septoplasty, and concluding that new treatment options need to be explored (see for review, Sund & Sunnergren, 2015; Harrill et al., 2007) [28,30]. From our point of view, there is not only a need of treatment but of diagnostic tests to objectively determine the exact point of obstruction, the relevance of the different structures involved in the nasal flow, and more important, the chance to perform a virtual surgery for, at least, to get a symmetric and inside-the normal-range nasal flow. In our experience, this goal can be achieved using CFD techniques, and it quite evident how all the six surgeons could, after the second round of surgeries, get a normal and symmetric flow in the two virtual cases.

When examining Table 2, Table 3, which detail the surgical procedures conducted by six otolaryngologists on two patients before and after receiving Computational Fluid Dynamics (CFD) feedback, certain trends become evident. In the pre-feedback round of surgeries, the diversity in surgical strategies is apparent, with each ENT making unique decisions based on their professional judgement. For instance, ENT 1 for Patient #1 performed anterior septoplasty work and middle third septal spur exeresis to right nasal fossa, while ENT 4 performed surgery to correct the posterior septal crest in area III-IV and reduced both turbinate heads. Such variability in surgical techniques may contribute to inconsistent postoperative outcomes, affecting the patient's quality of life.

However, in the post-CFD feedback round, a reduction in this variability is evident, suggesting the beneficial role of CFD feedback in standardizing surgical procedures. Notably, all ENTs, except ENT 5 for Patient #1 and ENT 4 and 5 for Patient #2, modified their surgical plans after receiving CFD feedback. This shows the capacity of CFD feedback to inform and refine surgical decisions, helping achieve better patient outcomes. For instance, ENT 1 for Patient #1 revised the previous surgery, and ENT 2 increased the small anterior septal deviation correction and completed the volumetric reduction of both inferior nasal turbinates. These modifications indicate a targeted response to the feedback, suggesting an increased focus on optimizing nasal airflow.

The use of CFD feedback also seemed to guide the surgeons towards particular areas within the nasal cavity. For instance, ENT 1 for Patient #1 focused in minor revisions of the previous surgery, whereas ENT 6 for Patient #2 focused on better realignment in the anterior septal area (area 2). This shift in focus after CFD feedback implies that the feedback provided specific, actionable insights that helped the surgeons understand which areas required more attention.

Interestingly, not all surgeons made modifications to their surgical plans after receiving the CFD feedback. ENT 5, in the case of Patient #1, and ENT 4 and ENT 5, in the case of Patient #2, stayed consistent with their initial surgical strategies. This suggests that their initial plans were in harmony with the insights provided by the CFD simulations, indicating that their original assessments matched the optimal airflow patterns proposed by the computational fluid dynamics analysis.

Another key observation relates to the change in surgical strategies from unilateral to bilateral procedures, or vice versa. For example, ENT 3 for Patient #1 transitioned from a unilateral approach to bilateral turbinoplasty after receiving CFD feedback. This transformation indicates the valuable insights provided by the CFD simulations about airflow dynamics, influencing the surgeon to modify their approach for improved outcomes.

A noteworthy pattern post-CFD feedback was the adjustments made to the extent of turbinoplasty by some surgeons. In Patient #2, ENT 3 initially performed minimal right turbinoplasty, but opted for extensive right turbinoplasty after receiving the feedback. This change reaffirms that CFD simulations provided essential information regarding the need for more extensive work to optimize nasal airflow.

It's intriguing to note a consistent trend among all six otolaryngologists across both patients; after receiving feedback from the Computational Fluid Dynamics (CFD) analysis on their initial surgery, all surgeons appear to adjust their surgical strategy in a way that aligns more closely with the parameters of the high-probability square [20]. This means that their post-feedback surgical interventions seem to steer towards the characteristics of a “healthy” nasal cavity as suggested by that high-probability area. This observation not only underscores the potential of CFD-guided feedback in promoting more standardized and effective surgical strategies but may also serve as an additional affirmation of the relevance and predictive power of the high-probability square concept. The tendency for surgical outcomes to gravitate towards this square after CFD-guided feedback could suggest that the definition of a healthy nasal cavity is being further substantiated by this research.

The utilization of CFD feedback appears to facilitate a shift towards standardized surgical outcomes by reducing variability in surgical approaches. This reduction in variability, coupled with the convergence of surgical outcomes towards the high-probability square, emphasizes the potential of CFD-guided feedback in improving the effectiveness of nasal surgeries, leading to more physiological nasal cavities and improved patient quality of life. The feedback not only improves the precision of surgical interventions but also reaffirms the relevance and predictive power of the high-probability square concept, making it a beneficial tool for ENT specialists in their quest for optimal patient outcomes.

There are some considerations to address in the current work. First, the aim of the surgeons was not to improve the patient symptoms, but to create a radiological ‘normal’ nasal cavity in a patient suffering from nasal obstruction. In this context, Lepley et al. (2023) [31] in their survey of 60 otolaryngologists trying to choose the patients complaining of nasal obstruction between CT scans of 10 confirmed symptomatic patients and 36 healthy controls, they found that on average, they identified 64.2 ± 29.8% of symptomatic NSD subjects correctly, but misidentified 54.6 ± 34.6% of HC as symptomatic. It is clear that in surgery for nasal obstruction the patient profile and clinical complains a proper selection of the case is mandatory [32]. Second, it is clear from our results, that a radiological satisfactory result does not imply a normal functional nasal cavity in CFD terms. In this context, CFD based surgery arises a novel instrument to improve surgical results, as the second round results demonstrate.

Moreover, researchers should investigate the potential for CFD analysis and 3D virtual surgery techniques to be applied to other areas of otolaryngology, such as sinus surgery, laryngeal procedures, or even pediatric cases. This could help to further establish the value of these advanced techniques in a broader range of surgical contexts. Additionally, future studies should consider the potential for integrating other forms of technology, such as artificial intelligence or machine learning, into the surgical planning process. These tools could potentially enhance the accuracy and efficiency of CFD analysis and 3D virtual surgery techniques, ultimately contributing to further improvements in patient outcomes and the standardization of surgical practices.

Despite the potential of CFD and virtual surgery to improve variability in nasal surgery, it is crucial to recognize that they should complement, not replace, a surgeon's experience and clinical judgment. As technology advances and novel tools for surgery simulation and planning emerge, we can anticipate a further reduction in nasal surgery variability and improved patient outcomes.

Variability in nasal surgery is a prevalent issue resulting in significant failure rates and unsatisfactory patient outcomes. CFD simulations and virtual surgery, alongside other emerging technologies such as 3D printing and virtual reality (VR), have the potential to reduce this variability and enhance surgical accuracy. The integration of these tools into surgical practice can improve education, communication, and patient satisfaction, ultimately contributing to better decision-making and patient outcomes. As technology continues to progress and new tools for surgery simulation and planning are developed, we can anticipate further reductions in nasal surgery variability and improved patient outcomes. It is crucial to recognize that these tools should complement, not replace, a surgeon's experience and clinical judgment. Collaboration between various stakeholders will be essential in ensuring the successful integration of these technologies into clinical practice and fostering further innovation in the field of otolaryngology.

## 5 Conclusions

This study represents a pioneering exploration into the application of Computational Fluid Dynamics (CFD) and 3D virtual surgery in otolaryngology, particularly focusing on nasal surgery. Our findings, based on a limited but insightful dataset involving two patients and six otolaryngologists, highlight the significant role these technologies play in reducing variability in surgical strategies and improving patient outcomes.

Key results indicate a marked decrease in the variability of critical parameters (QR, QL, Ф, and R) post-CFD feedback, demonstrating the potential of CFD and 3D virtual surgery in enhancing consistency in surgical decision-making. This consistency is critical in achieving optimal patient outcomes and indicates a move towards more standardized surgical approaches.

Moreover, the study underscores the potential of these technologies in personalizing surgical strategies to match individual patient anatomies, thereby enhancing the overall efficacy of nasal surgeries. Beyond clinical applications, the educational value of CFD and 3D virtual surgery as tools for training otolaryngologists is noteworthy, suggesting benefits in the broader scope of ENT specialist training.

While our study is an initial foray and is limited by its small sample size, its implications are significant, setting a foundation for future, more extensive research. Further investigations with larger patient cohorts are necessary to validate and expand upon these initial findings and to fully establish the role of CFD and 3D virtual surgery in otolaryngology. The expansion of this research is essential to fully realize the potential of CFD and 3D virtual surgery techniques in standardizing surgical outcomes and enhancing patient care in otolaryngology.

## Funding

This publication is part of the R&D project PID2019-105097RB-I00, funded by MCIN/AEI/10.13039/501100011033.

## Data availability statement

Data relevant to this study are not stored in publicly available repositories. Data will be made available on request. Data included in article/supp. material/referenced in article.

## CRediT authorship contribution statement

**M.A. Burgos:** Writing – review & editing, Writing – original draft, Visualization, Validation, Supervision, Software, Project administration, Methodology, Investigation, Funding acquisition, Conceptualization. **Lina Rosique:** Methodology, Investigation, Conceptualization. **F. Piqueras:** Methodology, Investigation, Conceptualization. **C. García-Navalón:** Methodology, Investigation, Conceptualization. **M.A. Sevilla-García:** Methodology, Investigation, Conceptualization. **D. Hellín:** Methodology, Investigation, Conceptualization. **F. Esteban:** Writing – review & editing, Writing – original draft, Validation, Methodology, Investigation, Funding acquisition, Formal analysis, Conceptualization.

## Declaration of competing interest

The authors declare the following financial interests/personal relationships which may be considered as potential competing interests:

Manuel Antonio Burgos Olmos reports financial support was provided by Spanish Ministry of Science and Innovation - PID2019-105097RB-I00. Dr. Manuel A. Burgos is the developer of Flowgy®. No other conflicts of interest exist, nor financial disclosure reported. If there are other authors, they declare that they have no known competing financial interests or personal relationships that could have appeared to influence the work reported in this paper.
